# Megafaunal Rodents: Behaviour and Ecological Roles of Southeast Asian Forest Porcupines

**DOI:** 10.1002/ece3.72552

**Published:** 2025-12-09

**Authors:** Kim R. McConkey, Alicia Solana Mena, Param bin Pura, Charang Muhamad Tauhid bin Tunil, Husin Sudin A/L Din, Lisa Ong, Ahimsa Campos‐Arceiz, Sean Eeshwaran Sinnaveeran, Sanjay D. Ramarao, Ee Phin Wong

**Affiliations:** ^1^ BIOTEC National Science and Technology Development Agency Khlong Nueng Thailand; ^2^ School of Biological and Environmental Sciences (Previously the School of Environmental and Geographical Sciences) University of Nottingham Malaysia Semenyih Malaysia; ^3^ Management & Ecology of Malaysian Elephants (MEME) University of Nottingham Malaysia Semenyih Malaysia; ^4^ Southeast Asia Biodiversity Research Institute, Chinese Academy of Sciences & Center for Integrative Conservation, Xishuangbanna Tropical Botanical Garden Chinese Academy of Sciences Mengla Yunnan China; ^5^ Yunnan International Joint Laboratory of Southeast Asia Biodiversity Conservation & Yunnan Key Laboratory for Conservation of Tropical Rainforests and Asian Elephants Mengla Yunnan China; ^6^ Community and Programmes Coordinator, Eats, Shoots & Roots Kuala Lumpur Malaysia; ^7^ Burgmann College Canberra Australia

**Keywords:** *Atherurus macrourus*, burrows, caching, *Hystrix brachyura*, seed dispersal, seed predation

## Abstract

Porcupines are the megafauna of Southeast Asia's rodent community and—as such—potentially perform important ecological roles in the rainforest habitats they are common in. We investigated four ecological roles of Malayan porcupines ( *Hystrix brachyura* ) and brush‐tailed porcupines ( *Atherurus macrourus* ) within the Belum–Temenggor rainforest complex of Peninsular Malaysia. First, camera traps placed at four Malayan porcupine and one brush‐tailed porcupine burrow showed that the burrows were used in varying ways by at least 22 other animal species. These animals shared the burrow, fed on insects at the entrance or possibly investigated the burrows as predators. Second, seedling surveys on top of the burrows, suggested that burrows might also be good microsites for seedling establishment and growth (as found previously in arid and semi‐arid habitats), with a higher species richness and density on the burrows than at control sites. Third, porcupines consumed 80 plant species (identified through Local Ecological Knowledge, or LEK, from the Indigenous community), 65% of which they acted as seed predators for. Fourth, they were seed dispersers of 33% of consumed species—mainly by hoarding—but also dispersed a few species by endozoochory or consuming only the pulp. The dispersed species (identified through LEK) included many megafaunal‐syndrome species, and porcupines also showed high dispersal overlap with elephants (and rats). Hence, as common, megafaunal rodents, porcupines are performing important ecological roles within rainforests. These findings highlight the important, yet overlooked, role of porcupines in maintaining tropical rainforest function, underscoring that their conservation is essential for sustaining tropical biodiversity.

## Graphical abstract

Southeast Asian porcupines (Malayan porcupine, *Hystrix brachyura* ; brush‐tailed porcupine, *Atherurus macrourus* ) performed at least four important ecological roles in a Malaysian rainforest. Burrows of both species were used in several ways by at least 22 animal species, while the soil above was potentially good sites for seedling recruitment. Porcupines also acted as seed predators for 51 plant species and seed dispersers for 26 species, especially megafaunal‐syndrome species.

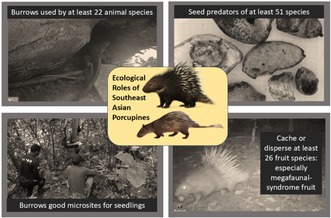

## 1 Introduction

Old World porcupines (Family: Hystricidae) are ubiquitous rodents within diverse habitats across Africa and Eurasia (Wilson et al. 2016). Porcupines are among the ‘megafauna’ of the rodent community, with some species weighing nearly 30 kg (Wilson et al. 2016). Their large size, high abundance, and fossorial habits have earned porcupines the label of ‘ecosystem engineers’ in arid and semi‐arid environments, where porcupine burrows provide essential shelter for many other animal species (Mori and Menchetti 2019; Mukherjee et al. 2019), and digging improves microhabitat conditions conducive for seedling growth as well as geophyte diversity (Bragg et al. 2005; Gutterman et al. 1990). Porcupines are also common consumers of seeds (Ong et al. 2022; Sidhu and Datta 2015; Velho et al. 2009) and can be among the most important seed predators within communities (K. R. McConkey unpublished data), thereby potentially playing pivotal roles in plant recruitment and the maintenance of plant diversity through seed predation (Paine and Beck 2007). Less frequently, porcupines have been observed to disperse seeds and spores by endozoochory and scatter hoarding (a form of synzoochory, whereby seeds are stored singly, and often germinate when the animal fails to retrieve them; Gómez et al. 2019); (Chaudhary et al. 2015; Ori et al. 2018; Mori et al. 2017; Rosin and Poulsen 2017). Despite their importance, the few studies investigating porcupine ecological functions are focused mainly on dry and seasonal habitats, such as deserts, grasslands and dry forests (e.g., Mori et al. 2017; Mukherjee et al. 2019; Gutterman et al. 1990), or on the wider animal community (e.g., Sidhu and Datta 2015; Nelabali 2023). Major gaps in our knowledge remain, especially concerning the ecological roles they perform in rainforest habitats.

Rainforests present very different environments for porcupines and the species they interact with, when compared to the drier habitats from which most studies have been conducted. In rainforests, temperatures are less variable and moisture more available (Woodward et al. 2004), so that dependence on burrows by nonfossorial species (e.g., Andersen et al. 2021; Dawson et al. 2019; Di Blanco et al. 2020) might be reduced compared to species in drier or seasonal habitats. Furthermore, the moisture‐laden depressions created by diggings that support seedling establishment in dry habitats (Bragg et al. 2005; Gutterman et al. 1990), might be less important in rainforests. While porcupine burrow distribution has been investigated in rainforests (Marina and Zubaid 2015), there is no available information on burrow use by other animals, and burrow‐associated seedling patterns, to evaluate whether these ecological roles considered important in drier habitats might also occur in rain forests. Given how common porcupines are in many rainforest habitats (Ahmad and Gopi 2024; Akash et al. 2025; Jambari et al. 2023; Vinitpornsawan and Fuller 2023), identifying their interactions with other species is important for understanding their potentially widespread impacts on ecosystems—impacts and functions that can potentially be lost with the ongoing decline of many rainforest‐dwelling porcupines (IUCN 2025).

Fruit and seed foraging habits of porcupines have been documented in both wet and dry habitats. Community‐level studies have identified porcupines as common seed predators (Sidhu and Datta 2015; K. R. McConkey unpublished data), but seed dispersal by endozoochory has been confirmed only for small seeds in arid and semi‐arid habitats (Chaudhary et al. 2015; Mori et al. 2017). Conversely, scatter hoarding has only been observed in two rainforest habitats, to varying extents (Gabon, Rosin and Poulsen 2017; Indonesia, Nelabali 2023) and no hoarding has been documented in some regions despite intensive studies (North‐east India, Sidhu and Datta 2015). There are no confirmed reports of larder hoarding by porcupines, although they use complex burrow systems (Marina 2019; Marina and Zubaid 2015); however, Phillipps and Phillipps (2016) speculate that some of Borneo's large, hard seeds might be larder‐hoarded by porcupines, and germinate within the burrows. Given the porcupines' large body size, it could be expected they would interact more frequently with the larger fruits and seeds in the forest and—as suggested by Phillipps and Phillipps (2016)—might have a tendency to also disperse these species. This could include the largest‐fruited species, called ‘megafaunal‐syndrome fruits’ or ‘megafruits’ whose large size (> 40 mm wide) limits the number of available dispersers (Guimarães et al. 2008; McConkey, Campos‐Arceiz, et al. 2022). Understanding the traits that determine whether seeds are only consumed or also dispersed can define the roles of porcupines more accurately. Furthermore, to encapsulate their seed predation and seed dispersal roles within communities it is necessary to understand the extent to which the plant species consumed are also dispersed or consumed by other animal species.

Four species of porcupines are known to occur in Malaysia. The Malayan ( *Hystrix brachyura* ) and long‐tailed porcupines ( *Trichys fasciculata* ) are widespread, occurring in both Peninsular Malaysia and the Malaysian states of Sabah and Sarawak on the island of Borneo (Wilson et al. 2016). The brush‐tailed porcupine ( *Atherurus macrourus* ) is confined to the Peninsula, while the thick‐spined porcupine ( *Hystrix crassispinis* ) is known only from Borneo. All three species in Peninsular Malaysia are protected by the Wildlife Conservation Act (Gomez and Min Sheng 2024). In the rainforests of the Belum–Temenggor Forest Complex, Peninsular Malaysia, two species of porcupine occur. Malayan porcupines are the largest (8–27 kg) and most commonly observed (L. Ong unpublished data; Shahfiz et al. 2008), while brush‐tailed porcupines are smaller and less common (hereafter brush‐tailed porcupine; 1–4.3 kg) (Wilson et al. 2016). Neither species is currently considered threatened, but both populations are believed to be decreasing due to hunting (for bezoars, meat and quills, Gomez and Min Sheng 2024; Loke et al. 2020) and habitat loss (IUCN 2025). These two porcupine species have shared the forest for millennia with the Indigenous Orang Asli (‘first’ or ‘original’ people), and can still be legally hunted by them (Loke et al. 2020). The forest complex has nearly 7000 resident Orang Asli, from the Jahai and Temiar groups (Lim et al. 2019). Both the Jahai and Temiar hunt and gather in the forest to some extent, with swidden agriculture being more predominantly undertaken by the Temiar (Loke et al. 2020).

In this study, we investigated three major components of the porcupine's potential ecological roles—porcupine burrows as a resource for other animals, and as a good microsite for seedling growth, and porcupines as consumers and dispersers of seeds. We also report basic behavioural data on the two porcupine species. Data were collected in the field and by recording Local Ecological Knowledge (LEK) of the Jahai and Temiar groups. We addressed the following questions: (1) What animal species interact with porcupine burrows? (2) Do burrows support a higher diversity and density of seedlings, compared to control sites? (3) Do burrows support a higher density of seedlings from consumed species (following Phillipps and Phillipps 2016), compared to control sites? (4) Are seeds of hoarded species larger and harder, than those species with consumed seeds? (5) Which animal species do porcupines overlap with the most, in terms of seed species consumed or dispersed? We also (6) determined the activity period of each porcupine species, to provide basic ecological information on this understudied family.

## 2 Methods

### 2.1 Study Site

This study was conducted in Temenggor (5°27′N 101°22′ E), the southern part of the Belum‐Temenggor Forest Complex (BTFC), Perak, Peninsular Malaysia. At around 3000 km^2^, BTFC is an important segment of Malaysia's Central Forest Spine (CFS; e.g., de La Torre et al. 2019), an Environmentally Sensitive Area (ESA) under the National Physical Plan (NPP) (Official Portal of JPSM 2022; Ramli et al. 2024). The humid tropical rainforest has a mean daily temperature of 24.3°C, ranging from 20.8°C to 33.5°C, and an average annual rainfall of 2700 mm. The forest complex extends across elevations ranging from 130 to 2160 m, comprising a heterogeneous assemblage of forest types, along with a mix of hill dipterocarp forest, lowland dipterocarp, upper dipterocarp and montane forests (Hanis et al. 2014). It forms a transboundary landscape with Bang Lang National Park and Hala Bala Wildlife Sanctuary, Southern Thailand. The north of BTFC is the Royal Belum State Park, encompassing 115 km^2^, representing the second largest protected area in Peninsular Malaysia (Chye 2010). By contrast, the Temenggor forest in the south is designated as a production Forest Reserve, comprising selectively logged patches of varying ages. The damming of BTFC's lowland areas created Tasik Temenggor, an artificial lake of 180 km^2^ hectares, interspersed with hundreds of islands (Ramli et al. 2024). BTFC harbours remarkable biodiversity, with around 3500 seed‐bearing plant species, in addition to over 80 mammalian species, among them several globally threatened taxa, including the Malayan tiger ( *Panthera tigris* ), Asian elephant ( *Elephas maximus* ), white‐handed gibbon ( *Hylobates lar* ), Malayan tapir ( *Tapirus indicus* ) and Malayan sun bear ( *Helarctos malayanus* ). Avifauna diversity is similarly high, with an estimated 300 bird species, inclusive of all 10 hornbill species recorded in Peninsular Malaysia (Ramli et al. 2024; Yeap et al. 2005).

This region is notable as the traditional territories of two indigenous Orang Asli communities: the Jahai and the Temiar (Lim et al. 2017). For livelihood, some have integrated with the mainstream work force (e.g., working in factories), while others carry out small‐scale rubber planting, cultivation (e.g., cassava), and traditional practices such as gathering of forest products like roots, wild fruits, honey, rattans and resins, as well as selective hunting of animals such as burrowing and arboreal species, and fishing (Aweng et al. 2020; Loke et al. 2020).

### 2.2 Overview of Field Methods

There are three components to this study: (1) placement of camera traps outside the burrows of both porcupine species, to document porcupine behaviour, use of burrows by other species and potential larder hoarding activities; (2) assessment of seedling diversity and abundance on top of Malayan porcupine burrows in comparison to control sites; and (3) documentation of consumed fruit, fruit traits, and fruit handling behaviours (seed hoarded, seed consumed, seed swallowed) by porcupines (at the Family level, Hystricidae) as described by the Orang Asli residing in the forest complex (LEK). Components (1) and (2) were limited by the COVID‐19 pandemic, which restricted access to the study area for almost the entire research period. Camera traps were maintained, when possible, during this period by the authors Param bin Pura, Charang Muhamad Tauhid bin Tunil and Husin Sudin A/L Din who lived in the region; however, we were unable to expand the sample sizes for either component. Component (3) was collected prior to the camera trap study (Ong et al. 2021, 2022); this dataset contains information on 34 animal taxa (documented at species, genus or family level), allowing a comparison with porcupines. Several aspects of the study (described below) rely on the expert knowledge of the authors Param bin Pura, Charang Muhamad Tauhid bin Tunil and Husin Sudin A/L Din who are from the Temiar group of Orang Aslis.

### 2.3 Camera Trap Observations Outside Porcupine Burrows

Burrows were monitored intermittently from September 2019 to January 2021. In late 2019, we searched for burrows of Malayan and brush‐tailed porcupines using two methods: knowledge of the Temiar resident in the region (as communicated through the Temiar authors), and searches in areas in which we had seen porcupines on cameras set for other studies. We were unsuccessful in finding any burrows by searches, however, and all burrows were located using personal knowledge of their location. We located five active burrow complexes within rainforest that were sufficiently accessible to be monitored and were hundreds of metres apart, one of which was of the brush‐tailed porcupine and the remainder were of Malayan porcupines; Appendix 2. The brush‐tail burrow and one Malayan porcupine burrow were in rockfaces, and the remaining Malayan porcupine burrows were in soil.

Cameras (model: Trophy Cam HD Bushnell, 6 units; Ereagle E1C Trail Camera, 1 unit) were attached to trees positioned appropriately to record activity outside the burrow entrances. Cameras were set to record 30 s videos, with minimum delay between captures and were active for periods ranging from seven to 85 days (median = 43 days) (Appendix 1), with the period determined entirely on our ability to access the sites during the COVID‐19 pandemic (hence, cameras were not checked within these periods). The burrow complexes of porcupines typically have multiple entrances (at least within soil substrates; Marina 2019). Our cameras were positioned at what we predicted to be a commonly used burrow entrance of each complex (i.e., one camera per burrow complex, which can typically cover 14 × 15 m; Marina 2019). Although we initially planned to move the cameras to a different entrance if few porcupine captures were observed, we were unable to return to the burrows regularly due to pandemic restrictions. As a result, the soil burrows had varied success in documenting porcupines, while we yielded more consistent results at the rockface burrows for which we found a single major entrance large enough for the porcupines (Appendix 1); note that it is possible other entrances were not visible to us. On two occasions, we left some large forest fruits (~15 fruits of *Callerya atropurpurea*; Fabaceae; Appendix 2) close to the burrow entrance to observe the treatment of the fruits by porcupines.

From the recorded videos and photographs, we noted times of activity of the porcupines, the number of individuals and whether they were adult‐sized, subadults (slightly smaller than adults) or young. We also recorded the identity of other species observed to interact with the burrows (entered/exited burrow; investigated the entrance; ate insects at entrance), to the lowest taxonomic level possible; we did not include videos of animals that passed by with no interaction. Finally, we recorded any interesting behaviour ad libitum, including whether the porcupine carried items into the burrows. If more than 15 min separated two observations, we arbitrarily recorded the visits as separate observations; however, this does not reflect independence given that we observed the same individuals but defines the temporal scope of the study.

### 2.4 Seedling Surveys on Burrows

Seedling surveys were conducted on top of six soil burrows of Malayan porcupines (three of which were also used for the camera trapping described above). We surveyed for seedlings in the areas in between burrow entrances (see Appendix 3 for distances and dimensions of searched plots, which varied according to burrow size). Seedling surveys over the burrows were matched by a survey of a similar area 10 m away from the burrow (control), in areas with similar slope and aspect as the burrow. An area of between 4.5 m^2^ and 10 m^2^ was surveyed for seedlings at each location (Appendix 3). We identified and counted all seedlings (< 1 m in height) over the burrow and in the control plots, but did not include seedlings of species from overhead plants. Plant species were identified by Param bin Pura, Charang Muhamad Tauhid bin Tunil and Husin Sudin A/L Din, using Temiar names. We also determined whether the fruit or seeds of the species were known to be consumed by porcupines, based on the expert knowledge of the Temiar authors, and LEK data collected by Lisa Ong (Ong et al. 2021).

### 2.5 Fruits Species Consumed and Handling Behaviour Determined by LEK

A full description of the methods to identify fruit consumed by porcupines can be found in Ong et al. (2021). In summary, a fruit catalogue (including photos of fruits, seeds and leaves) was developed from phenology surveys conducted in Royal Belum State Park (within the same forest complex) over 16 months. This catalogue, along with visual representations of a range of animal taxa and different fruit and seed handling behaviours, was presented in interviews with 30 participants from the Temiar and Jahai groups. Each individual or a pair of participants was asked to select the fruit species eaten by each animal taxon and to identify whether the species was hoarded, dispersed via endozoochory or stomatochory, or if the seed was destroyed. Hence, the interviews provided information on seed consumption and seed dispersal. These interviews build on a deep link between the Orang Asli and their knowledge of the ecosystem they inhabit (i.e., both LEK and TEK, or Traditional Ecological Knowledge). In total, the interviews collected information on 164 plant species and 34 animal taxa. Many animal taxa were not investigated to species level to avoid over‐burdening the interviewees. In the case of porcupines, responses referred to the family level (Hystricidae) and we cannot determine differences between the two species. In the results here, we present data on the seed dispersal overlap between porcupines and other animals, based on data collected using the same methods and during the same time. Informed consent was obtained from all interviewees before proceeding. Further details on the use of LEK in Malaysian ecosystems can be found in Ong et al. (2022) and McConkey, Aldy, et al. (2022).

Fruit and seed traits were collected for the plant species collected during the phenology surveys and presented in the interviews described above. The traits measured were fruit width (mm), seed width (mm), fruit type (dry/wet), fruit hardness (easy, moderate, hard–to pierce the fruit), seed hardness (soft, soft‐medium, medium, medium‐hard, hard). We also noted the number of megafaunal‐syndrome plant species (fruit width > 40 mm; Guimarães et al. 2008; McConkey, Campos‐Arceiz, et al. 2022). Fruit hardness is categorised by how easily a fruit can be pierced and its overall structure. Fruits that are easy to pierce are usually soft with thin skins. Moderate fruits are firmer, often with rinds at least a millimetre thick. Hard fruits resist piercing and may need forceful cutting or a nutcracker. Seed hardness parallels this: soft seeds break with a pinch and have thin coats; soft‐medium needs more pressure or may be gritty small seeds; medium requires cutting or force, often with firm cotyledons or fibrous coats; medium‐hard demands cutting due to fibrous coats or ruminate endosperm; hard seeds typically require cutting or cracking with more force.

### 2.6 Data Analyses

All statistical analyses were completed on Python (version 3.12). The *pandas* package was used for data frame handling, and the *matplotlib* and *seaborn* packages were used for data visualisation. In the kernel density plots, the shaded time shows the dusk to dawn period recorded during the study period for northern Peninsular Malaysia. To test whether seedling density and richness (per unit area) were higher on burrows than at control sites, we used a one‐tailed Mann–Whitney *U* test. Similarly, we also used one‐tailed Mann–Whitney *U* tests to compare fruit and seed sizes between hoarded and consumed species, based on prior findings that rodents often hoard larger propagules (Vander Wall 2010). Chi‐squared tests were used to test for differences between hoarded and consumed species for categorical traits, and to assess whether megafruits were more likely to be hoarded or consumed exclusively.

## 3 Results

### 3.1 Burrow Overview

Between September 2019 and June 2020, and again in January 2021, we monitored six burrows of Malayan porcupines over a total of 260 camera‐trap days (range, 11–72 days per burrow; mean = 37 days), and one burrow of brush‐tailed porcupine over 216 days (Appendix 1). Two of the Malayan porcupine burrow entrances were mostly inactive. We recorded 189 independent visits of Malayan porcupines and 224 visits for brush‐tailed porcupines (each visit of one or more individuals). One of the Malayan porcupine burrows was at the base of a rockface, and the others were in soil with more than one entrance, making monitoring difficult. The brush‐tailed porcupine burrow was in a rockface, and we only located a single entrance. The brush‐tailed porcupine burrow had a latrine on the outer side of the rockface with a large collection of old faecal matter resembling porcupine faeces, but we could not confirm if this was generated by the porcupines or other inhabitants of the burrow.

For Malayan porcupines, all active burrows contained one young, and two of the five burrows also had one nearly adult‐sized individual, along with two adults. At one inactive entrance monitored we recorded a single adult. The brush‐tailed porcupine burrow had two adults and one young at the start of monitoring, as well as a subadult. By the end of monitoring at the brush‐tailed porcupine burrow we recorded six individuals in the burrow, all large in size.

### 3.2 Porcupine Activity Recorded Outside Burrows

**FIGURE 1:**
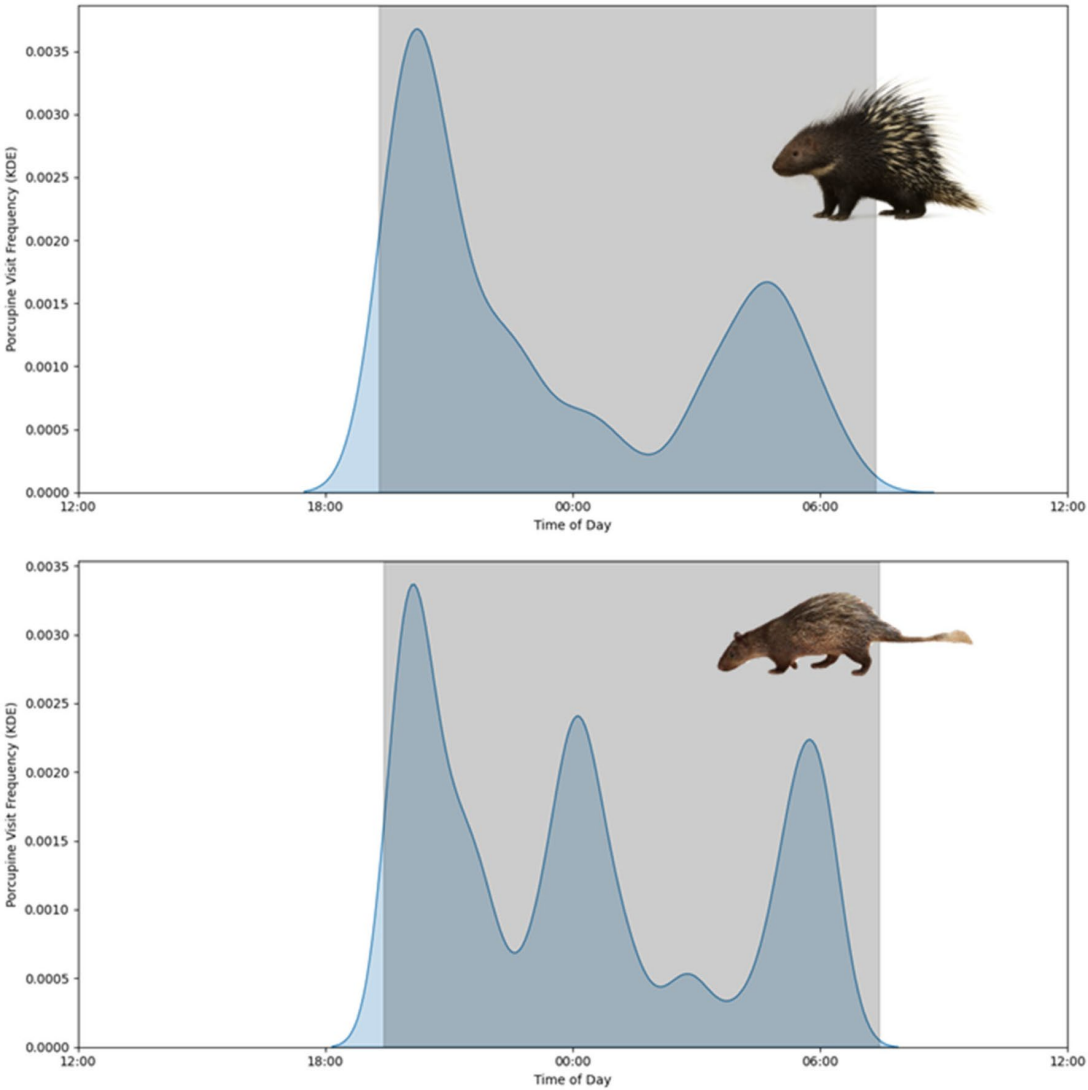
Activity period of *Hystrix brachyura* (top) and *Atherurus macrourus* (bottom) over 24 h. The kernel density plots illustrate temporal activity, with distributions centred around nighttime to reflect the species' nocturnal behaviour, with the shaded region showing dusk to dawn times at the time of study.

Both porcupine species left their burrows at dusk (Figure 1), but other activity was variable between species. Malayan porcupines returned mostly before dawn, and showed little burrow activity in the middle of the night. Brush‐tailed porcupines also returned before dawn, but had a third peak of activity in the cameras in the middle of the night (around midnight). Both species were rarely observed outside during the daylight hours.

Brush‐tailed porcupines spent more time engaged in activities directly outside the burrow entrance in a single recording (median = 5:59 min per recording; range of 00:46 min to 2 h 4:56 min, *n* = 104 observations), than Malayan porcupines (median = 3 min, range 00:40 to 23:00 min; *n* = 65 observations). These values exclude videos where the porcupines directly entered or exited the burrows and demonstrated no other activity at the burrow entrance (i.e., all observations under 30 s). Furthermore, we could not determine total time away from the burrows, since we could not recognise individuals and could not be certain they entered and exited by the same entrance. Little activity was observed for Malayan porcupines outside the burrow entrances, but for brush‐tailed porcupines we observed two mating events (Video S1), a grooming session (Video S2), and play (Video S3) near the burrow entrance. Over 3 days, we also observed the individuals chase, and prevent a subadult from entering the burrow. This behaviour involved foot stamping (Video S4) and active chasing (Video S5).

We observed 11 items being carried into the burrow by Malayan porcupines. Four were seeds of *Callerya atropurpurea* that we had left close to the burrow entrance. The porcupines removed the seeds from the pods before carrying the seeds singly into the burrow. Another large unidentified fruit was observed being carried into the burrow. Other items carried into the burrows were a large fungus (once) and sticks (four times). The only items brush‐tailed porcupines were observed carrying into the burrows were four large whole fruits (i.e., without removing the seeds) we left close to the entrance.

### 3.3 Visits to Burrows by Other Animals

**FIGURE 2:**
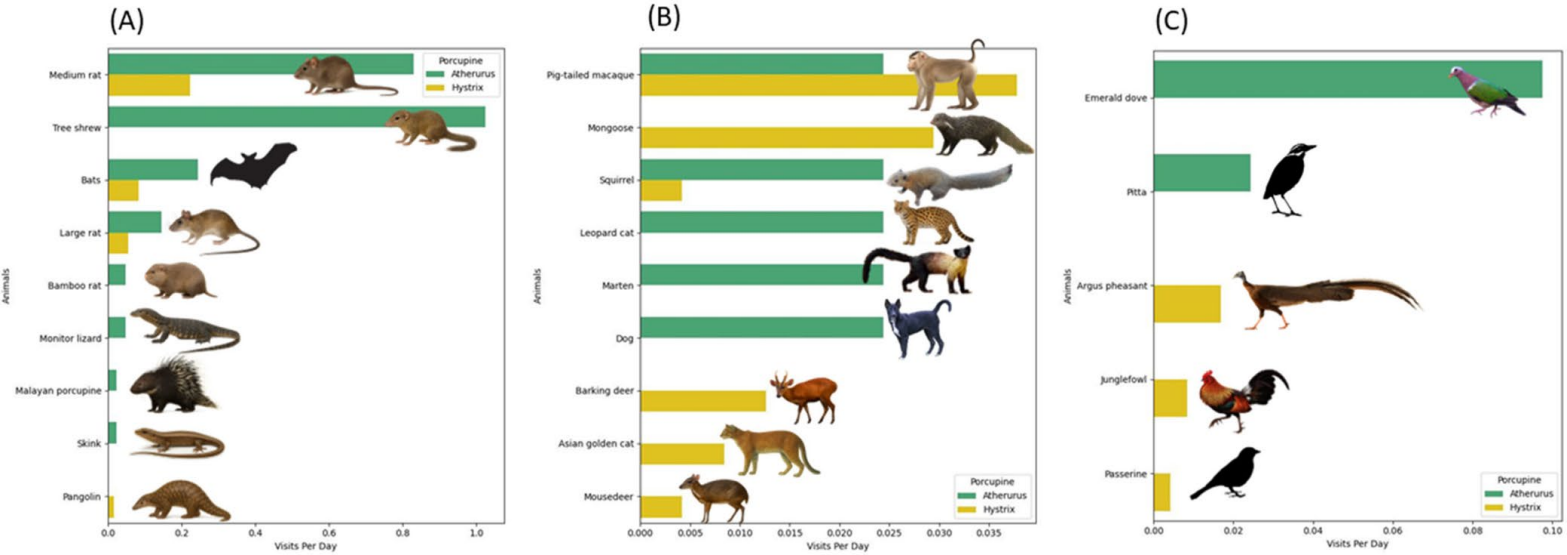
Animals seen visiting burrows of brush‐tailed porcupines (green) and Malayan porcupines (yellow), and the relative frequency of their visits (measured as visits per camera‐trap day). (A) animal species that entered the burrows; (B) animal species that investigated the burrow entrance; (C) animal species that fed on insects at the burrow entrance. See Appendix 4 for scientific names.

Overall, the brush‐tailed porcupine burrow in the rockface was interacted with by more animals (*n* = 14 species), than Malayan porcupine burrows (range of 1–5 species, median = 3) (Appendix 4). Most of the soil burrows of Malayan porcupines had more visitors (2–5 species) than the rockface burrow (1 species). We identified nine taxa that entered the burrow (Figure 2A); eight taxa were observed at the brush‐tailed porcupine burrows and four taxa at the Malayan porcupine burrows. Bats (unknown species), a medium‐sized rat (likely a species of *Maxomys* or *Rattus*) and a large rat (likely a species of *Leopoldalmys*) were seen at burrows of both species. One of the rats was seen carrying a fruit inside. Independent visits by two individual monitor lizards (*Varanus* sp.) showed them to exit or enter the brush‐tailed porcupine burrow, suggesting they used two entrances. The visit by Malayan porcupines to the brush‐tailed porcupine burrow was brief (6 min; Appendix 4).

Eight taxa investigated the burrow entrance but did not enter (Figure 2, Appendix 4). Five of these species are carnivores: domestic dogs ( *Canis familiaris* ), leopard cat ( *Prionailurus bengalensis* ), Asian golden cat ( *Catopuma temminckii* ), yellow‐throated marten ( *Martes flavigula* ) and crab‐eating mongoose (*Urva urva*) (Figure 2B). Five taxa—all birds—appeared to be feeding on insects (Figure 2C).

### 3.4 Seedlings on the Soil Burrows of *Hystrix*

**FIGURE 3:**
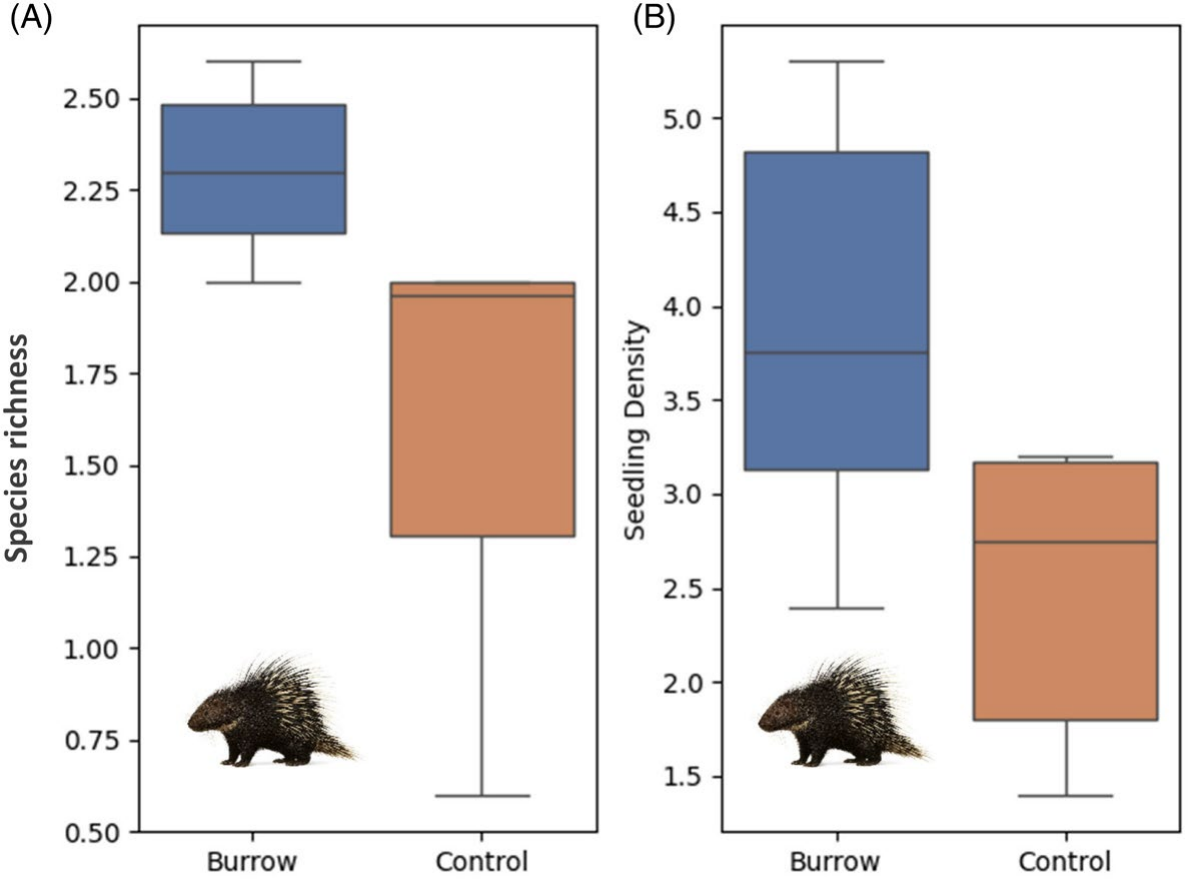
Species richness (A), and seedling density (B) growing on six porcupine burrows (soil burrows of Malayan porcupines), compared to a control site 10 metres away.

Species richness on the burrows (2.30 ± 0.24 species m^−2^) was higher than richness at the control sites 10 metres away (1.60 ± 0.61 species m^−2^) (Mann–Whitney *U* test, *U* = 34.5; *p* = 0.005) (Figure 3). A higher number of seedlings (1.4 times) was found on the Malayan porcupine burrows (mean ± SD = 3.88 ± 1.15 seedlings m^−2^) than off the burrows (mean ± SD = 2.5 ± 0.82 seedlings m^−2^) (*n* = 6 burrows; Mann–Whitney *U* test, *U* = 29.5; *p* = 0.039) (Figure 3; Appendix 3). There was no difference in the proportion of seedlings that were species of porcupine foods on burrows (mean ± SD; 0.25 ± 0.15 seedlings m^−2^), compared to the proportion at the control site (0.24 ± 0.15 seedlings m^−2^) (*U* = 17, *p* = 0.591).

### 3.5 Fruit and Seed Species Consumed by Porcupines Reported by LEK

Porcupines consumed fruits and/or seeds of 80 plant species. Of these, 51 species had the seeds consumed with no dispersal (65% of all eaten species), and 26 species (33%) had the seeds dispersed, either by endozoochory (five species, all figs) or hoarding (21 species) (Appendix 5). The porcupines ate the pulp of two species (*Bouea oppositifolia, Citrus hystrix*) and dropped the seeds under the parent crown, and the fate of one species was unknown.

**FIGURE 4:**
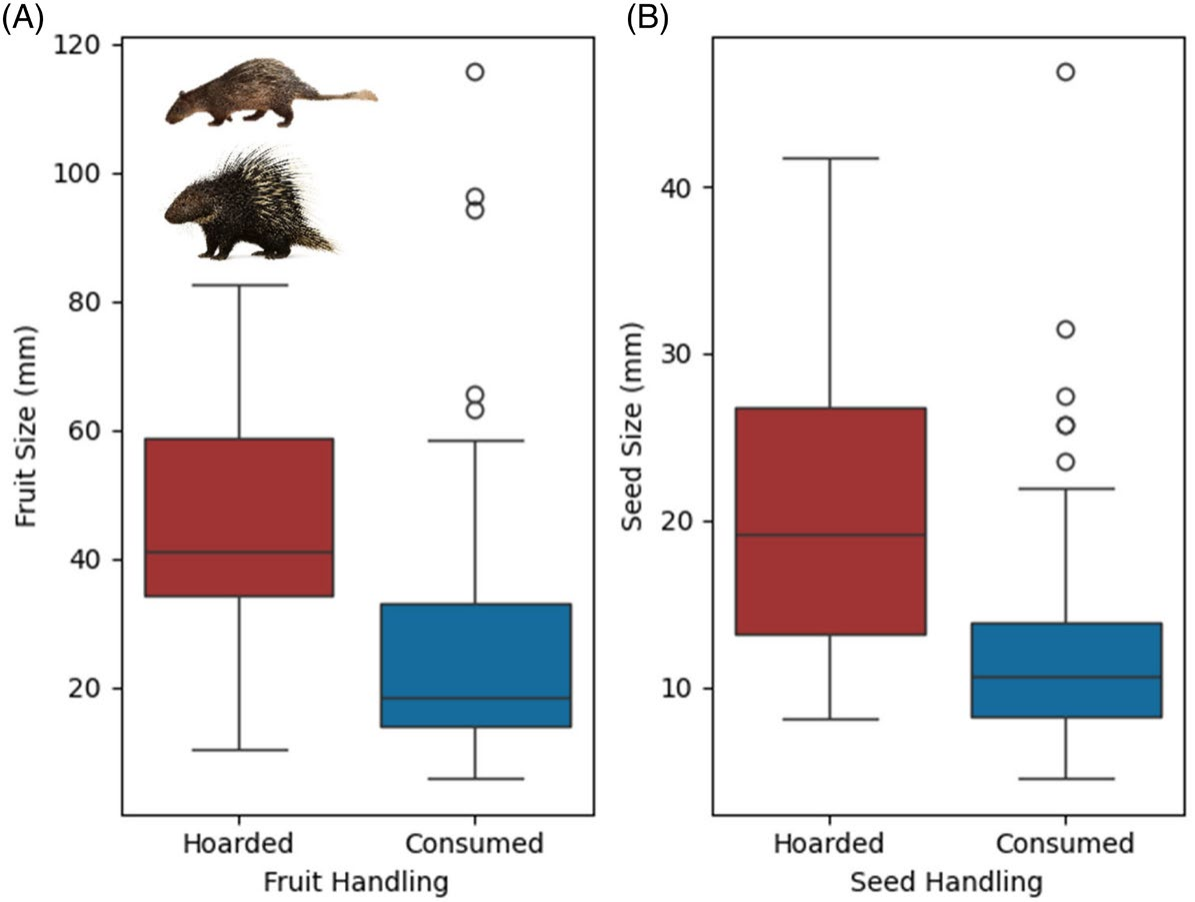
Size of fruits (A) and seeds (B) consumed by porcupines (Family: Hystricidae: Results are for both species combined) according to two types of seed handling behaviours—hoarded (red) and consumed (blue).

Fruits consumed by porcupines ranged from 5.8 to 115.7 mm in width (mean ± SD, 32.6 ± 24.1) and the seeds from 0.7 to 46.9 mm wide (14.3 ± 9.4). A third of the fruits (32.5%) were megafruits (> 40 mm wide). Seeds that were hoarded tended to be larger than seeds that were only consumed (*H* = 857, *p* = 0.00003) and were also from larger fruit (*H* = 813.5, *p* = 0.0003) (Figure 4). Of the megafruits dispersed, 55% of them were hoarded rather than consumed only, and the porcupines were significantly more likely to hoard megafruits than smaller fruits, which were predominantly consumed only (*χ* ^2^ = 14.561, *p* = 0.0001). About 29% of the fruits consumed were dry, rather than fleshy but the incidence of this did not differ between hoarded and consumed species (*χ* ^2^ = 0.476, *p* = 0.490). Fruit hardness was variable and did not differ between hoarded and consumed species (*χ* ^2^ = 1.860, *p* = 0.394). Most fruits had hard seeds (70%) and this tendency did not differ between seed handling types (*χ* ^2^ = 1.470, *p* = 0.225).

**FIGURE 5:**
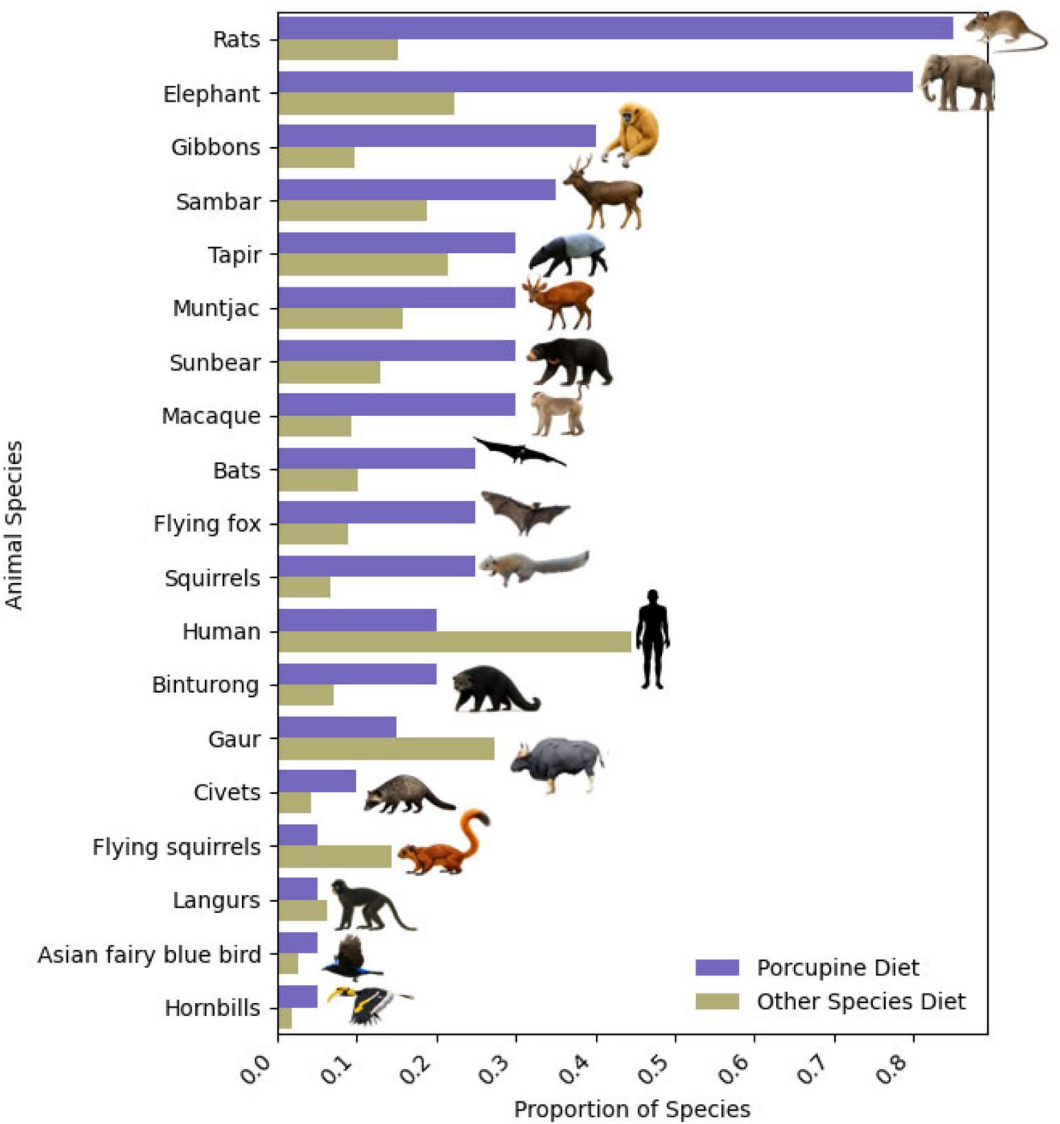
Proportion of plant species dispersed by nonporcupine species that overlap with that dispersed or consumed by porcupines. Purple bars show the overlap of species dispersed by porcupines that were also dispersed by other species, excluding *Ficus* species. Gold bars show the number of species dispersed by each animal taxa that are also consumed by porcupines. Purple bars show overlap from a porcupine's perspective and gold bars represent overlap from the other species perspective.

Porcupines shared a dispersal role (dispersed the same plant species) with 33 other animal species. Fourteen animal species (all birds) only had dispersal overlap for *Ficus* species, which are known to have many dispersers and are unlikely to represent similarity in preferences. Excluding the overlap for fig species, 19 animal species shared a dispersal role with porcupines (Figure 5); the highest overlap was with rats and elephants (seeds dispersed by porcupines, were most likely to also be dispersed by rats and elephants). Calculating from the perspective of the nonporcupine species, elephants still had among the highest overlap with porcupines (seeds dispersed by elephants, which are also consumed by porcupines), but humans, gaur, tapir and sambar also have a good proportion of dispersed species that are consumed by porcupines.

Porcupines were not identified to be the sole disperser of any species, but two plant species had only two dispersers (porcupines and rats: *Adinobotrys atropurpureus* (Fabaceae); porcupines and elephants: *Hydnocarpus* sp. 1 (Achariaceae)). Both these species had very hard megafruits (> 40 mm wide), and medium‐hard seeds (Appendix 5); Six species had three recorded dispersers (porcupines, elephants, rats: *Chisocheton* cf. *macrophyllus* (Meliaceae), *Intsia palembanica* (Fabaceae), *Saraca cf indica* (Fabaceae), *Scorodocarpus borneensis* (Olacaceae), *Eugeissona tristis* (Arecaceae); porcupines, rats, squirrels: an unidentified species). These six species also had megafruits, with medium to hard seeds (Appendix 5).

## 4 Discussion

Porcupines (Malayan and Brush‐tailed) perform important ecological roles in the rainforests they inhabit in Malaysia. We identified four ecological roles in this study, two of which have only previously been described in porcupines inhabiting arid and semi‐arid regions (Bragg et al. 2005; Gutterman et al. 1990; Mukherjee et al. 2019). Just as in these habitats, the rainforest porcupines shared burrows with at least nine other animal taxa, and the surface of soil burrows also proved to be hospitable environments for seedling establishment and/or growth. We also confirmed seed predation and seed dispersal roles for the porcupines (*n* = 80 plant species) and provided the first documentation that porcupines carry seeds into rainforest burrows, possibly reflecting limited larder‐hoarding behaviour. In particular, porcupines hoarded larger seeds and fruits—more often megafaunal‐syndrome species, some of which had only one or two alternative dispersers. The relatively high consumption/dispersal overlap that porcupines had with elephants, the preponderance of large fruits and seeds in their diets, and the potential (via shared burrows) and confirmed (microsites for seedlings) positive benefits brought by porcupine burrows, suggest this megafaunal rodent occupies a unique functional niche in rainforest habitats. Furthermore, in the context of ongoing defaunation of rainforest megafauna (Galetti et al. 2018), even the somewhat redundant roles could increase in importance in the future if they provide important back‐up dispersal roles.

### 4.1 Use of Porcupine Burrows by Other Animal Species

In arid and semi‐arid regions, porcupines are considered ecosystem engineers, partly because they create burrows that provide stable microclimates and predator refuges, thereby contributing to the survival of many other species (Mori and Menchetti 2019; Mukherjee et al. 2017, 2019)—a role confirmed for many fossorial species in harsh habitats (Andersen et al. 2021; Dawson et al. 2019; Di Blanco et al. 2020; Sun, Mao, et al. 2025; Sun, Zhang, et al. 2025). Here, we found similar associations in porcupine burrows in rainforest habitats, with at least 22 vertebrate species interacting with the burrows in multiple ways, and with the single rockface burrow of the brush‐tailed porcupine being used by more species than all of the Malayan porcupine burrows. However, as the first investigation of burrow sharing in a rain forest habitat, we cannot confirm whether the porcupines occupying the rockface burrows are supporting the presence of other species (as documented in arid areas) or if these are simply shared burrows with multiple animals maintaining the complex.

Of the nine species that entered and/or exited the burrow, seven species might inhabit the burrow or use it regularly. Tree shrews, bats, rats, bamboo rats, pangolins and skinks are all taxa known to inhabit burrows at times and some are also fossorial and might excavate their own burrow from within the complex (Alcala et al. 2004; Chao et al. 2020, Lim 2016, Mukherjee et al. 2019, Shadbolt 2014, Wells et al. 2006). Bats and skinks are probably underreported as they were usually observed with other animals, suggesting they did not trigger the camera, or—in the case of bats—had moved beyond the camera range between triggering and capturing the image. The reasons for using the burrows are potentially similar to those in harsher environments—refuge from predators, and shelter from weather. Monitor lizards and Malayan porcupines (at a brush‐tailed porcupine burrow) were observed entering the burrow: Monitor lizards also inhabit burrows (Traeholt 1995), but might prey on some of the inhabitants (Traeholt 1997), so it is not clear what their intent was in the two observations we recorded. Similarly, the brief visit of Malayan porcupines to the brush‐tailed porcupine burrow might represent an investigation to find a new burrow, or to raid food. Despite the diverse array of other animals using the burrows, the burrow entrances we monitored were primarily used by porcupines. However, all the burrows likely had other entry and exit points that we did not discover (Marina 2019) and some of the animals we recorded might use these, or smaller exit or entry points, and be permanent inhabitants of these burrows.

A different set of animals interacted with the burrows by either investigating the entrance, or, possibly consuming insects at the entrance. Five insectivorous or omnivorous birds (emerald dove, great argus, jungle fowl, unidentified pitta, unidentified passerine) were seen at the entrance and appeared to be feeding on insects—a behaviour seen at burrows of other species (Andersen et al. 2021). A leopard cat, Asian golden cat, crab‐eating mongoose, yellow‐throated marten and a domestic dog were also seen investigating the entrance and all are potential predators of some of the animals inhabiting the burrow (Kawanishi and Sunquist 2008; Rajaratnam et al. 2007; Subrata and Permatasari 2023); similarly, predators visit American badgers ( *Taxidea taxus* ) burrows, potentially to prey on animals using the burrows (Andersen et al. 2021). We cannot guess the intentions of the pig‐tailed macaques, unidentified squirrel, barking deer or mousedeer, which might also have been attracted by insects (Awasthi et al. 2024; Bernstein 1967) or were simply investigating the space. Hence, porcupine burrows possibly provide feeding opportunities for a range of different animal species than those that enter the burrow.

### 4.2 Seedlings Growing on Porcupine Burrows

The ground above the soil burrows of *Hystrix* were also potentially good environments for the establishment or growth of seedlings. Although our sample size (six burrows) was low we found a higher species richness and density of seedlings on the burrows than at control sites 10 metres away. A similar pattern was observed in a semi‐arid environment, in which the depression caused by the burrow holds more water in this water‐limited environment (Bragg et al. 2005; Gutterman et al. 1990). Our findings show that even in moist environments, such as rainforests, the benefit might still occur, with the turnover of soils possibly benefiting seeds that fall on top of the burrows in ways beyond simply water retention (Bragg et al. 2005; Gutterman et al. 1990). Alternatively, the burrows could be dug in more fertile soils, or topographic positions that favour plant establishment, and consequently, burrow construction is not the underlying reason for our results. We did not find evidence that species consumed by porcupines were more likely to grow on the burrows; this could suggest a low capacity for seeds hoarded by porcupines (or the rats they share the burrows with) to germinate (contrary to suggestions by Phillipps and Phillipps 2016), but we suggest a greater sample size is required to confirm these findings and for identifications to separate hoarded vs. consumed seeds (which was not done for the seedlings).

### 4.3 Seed Dispersal, Predation and Hoarding by Porcupines

Porcupines were confirmed by the Orang Asli to hoard seeds of around one third of the 80 plant species they consumed the fruits and seeds of. Unfortunately, the interviewees were not asked to distinguish larder hoarding from scatter hoarding, so we cannot identify which behaviour is more frequent, although the consequences for seeds can be quite different. Scatter hoarding (seeds buried singly and shallowly) is more likely to result in germinated seeds than larder hoarding (much larger collections, often buried deep in burrows) (Gómez et al. 2019). Our cameras confirmed that fruit and/or seeds were carried infrequently into burrows (along with sticks and fungi) and could have been either larder‐hoarded, or consumed immediately. Malayan porcupines have been observed larder hoarding the large seeds of the legume *Callerya atropurpurea* in the forest complex (Param bin Pura & Charang Muhamad Tauhid bin Tunil, personal observation). We camera‐trapped a tree of this species for 16 days and recorded 15 visits by Malayan porcupines; in all but one visit the porcupines' carried seeds or fruits away (*n* = 1–9 fruits per visit) from the tree, often removing the outer husk before doing so (Appendix 6). We suggest that the carrying of fruit/seeds represents hoarding as porcupines are more often recorded consuming fruits/seeds under the tree crown (L. Ong unpublished data; K. R. McConkey unpublished data revision; Nelabali 2023); given that the time span of removal was often quite short (e.g., 9 seeds in 10 min) we also suggest some of the seeds might have been scatter‐hoarded rather than carried back to the burrows. Interestingly, we found nine seedlings of this species growing on four Malayan porcupine burrows, and only three seedlings (at two control sites) off the burrows: This pattern could possibly be explained by germination of larder‐hoarded seeds (but requires a larger sample size to confirm), or possibly scatter hoarding close to the burrows. In some Asian forests, porcupines have not been confirmed to hoard seeds at all despite extensive studies (North‐east India; Velho et al. 2009; Sidhu and Datta 2015); however, the Sundaic forests where our study occurs have highly seasonal fruiting phenology (Van Schaik 1986) with extended periods of fruit shortages that negatively impact the fauna (Wong et al. 2005). We suggest that porcupines are more likely to hoard seeds in these forests (see Nelabali 2023). Further studies are required to determine how important these behaviours are for recruitment of the plant species hoarded.

Porcupines are the ‘megafauna’ of the rodent community in Asian rainforests. A third of the plant species identified to be consumed through LEK had megafruits with fruit width greater than 40 mm (Guimarães et al. 2008; McConkey, Aldy, et al. 2022) and megafruits had a higher tendency to be dispersed. Megafruits are considered to rely mainly on megaherbivores for seed dispersal, but our findings suggest porcupines—as a megafaunal rodent—might also be important for the recruitment of these species. Indeed, porcupines had among the highest dispersal and consumption overlap with elephants and the camera trapping of *Callerya atropurpurea* revealed that only elephants were a potential second, but infrequent, disperser of this megafruit (Appendix 6). The dispersal profiles of these two taxa are likely to be complementary rather than redundant due to presumed differences in dispersal distances and frequency of consumption. However, as megafauna, such as elephants, decline within Southeast Asian habitats, the role of porcupines in seed dispersal—particularly of megafruited species—could become increasingly important. Furthermore, just as megaherbivores perform important mutualistic (seed dispersal; McConkey, Campos‐Arceiz, et al. 2022; Tan et al. 2021) and antagonistic interactions (i.e., herbivory; Terborgh et al. 2025), porcupines are seed dispersers (Rosin and Poulsen 2017; Nelabali 2023) and dominant seed predators at the community level (McConkey et al., n.d. in revision)—an interaction that is pivotal to the maintenance of plant species diversity (Paine and Beck 2007). Porcupines also showed high dispersal overlap with rats, possibly reflecting the fruit types generally selected by terrestrial rodent species. Porcupines consume a range of other foods as well (bark, fungi) suggesting that their community‐level roles could be extensive and require more studies to fully assess. In European habitats, for example, porcupines also disperse fungal spores (Ori et al. 2018) and are implicated in the spread of an invasive species (Mori et al. 2017).

## 5 Conclusions

This paper describes some of the varied ecological roles of Southeast Asian porcupines, but more research is required to understand these roles more completely. We did not manage to obtain the sample sizes we initially aimed for, because of the COVID‐19 restrictions, and we had also planned to investigate scatter‐hoarding behaviours in more detail. Nevertheless, we provide clear evidence for four pivotal ecological roles of porcupines: porcupines are major seed predators, potentially contribute to seed dispersal via hoarding—especially of large‐seeded megafruits—provide shelter for other animal species within their burrows, and produce suitable microhabitats for seedlings. We encourage further studies in Asian rainforests to quantify the broader implications of these roles, and also encourage studies in the wet forests of Africa which present a similar habitat. Neither of the porcupine species studied here is currently considered to be threatened (IUCN 2025), but both have declining populations. When common species perform unique and important roles within an ecosystem, it is essential that such species remain common (Gaston 2010). Both species are hunted and traded as live animals and for bezoars, meat, and quills (Gomez and Min Sheng 2024; Loke et al. 2020) and their future as ‘common’ animals is at threat. We urge further work on these megafaunal rodents to uncover other important roles they perform, to better encapsulate their roles within ecosystems and to provide a factual basis for the need to ensure they remain common within the ecosystems in which they occur.

## Author Contributions

**Kim R. McConkey:** conceptualization (equal), data curation (equal), funding acquisition (equal), investigation (equal), methodology (equal), project administration (equal), visualization (supporting), writing – original draft (lead). **Alicia Solana Mena:** conceptualization (equal), data curation (equal), funding acquisition (equal), investigation (equal), methodology (equal), project administration (equal), writing – review and editing (supporting). **Param bin Pura:** conceptualization (supporting), investigation (equal), methodology (equal), project administration (equal), writing – review and editing (supporting). **Charang Muhamad Tauhid bin Tunil:** conceptualization (supporting), investigation (equal), methodology (equal), project administration (equal), writing – review and editing (supporting). **Husin Sudin A/L Din:** conceptualization (supporting), investigation (equal), methodology (equal), project administration (equal). **Lisa Ong:** conceptualization (supporting), data curation (supporting), funding acquisition (supporting), investigation (supporting), methodology (supporting), project administration (supporting), writing – review and editing (supporting). **Ahimsa Campos‐Arceiz:** conceptualization (supporting), funding acquisition (supporting), project administration (supporting), supervision (supporting), writing – review and editing (supporting). **Sean Eeshwaran Sinnaveeran:** data curation (equal), writing – review and editing (supporting). **Sanjay D. Ramarao:** data curation (supporting), formal analysis (lead), visualization (lead), writing – review and editing (supporting). **Ee Phin Wong:** project administration (equal), resources (equal), supervision (equal), writing – review and editing (equal).

## Conflicts of Interest

The authors declare no conflicts of interest.

## Supporting information


**Video S1:** Mating episode with Asiatic brush‐tailed porcupine, *Atherurus macrourus*, outside burrow.

**Video S2:** Grooming episode with Asiatic brush‐tailed porcupine, *Atherurus macrourus* .

**Video S3:** Play between two Asiatic brush‐tailed porcupines, *Atherurus macrourus* .

**Video S4:** Foot stamp by Asiatic brush‐tailed porcupine, *Atherurus macrourus* .

**Video S5:** Chasing of a presumed subadult Asiatic brush‐tailed porcupine, *Atherurus macrourus*, from the burrow.

## Data Availability

All data files are provided for review purposes. Data for the published manuscript will be available via a Dryad repository.

## Appendix 1

Details of each burrow that was camera‐trapped. Porcupine records refer to the number of independent recordings of porcupines (videos > 15 min apart). The number of individuals in the burrow is the highest number of individuals recorded throughout the monitoring period (the number of adults, the number of subadults, the number of young). Species of other animals are those that interacted with the burrow in some way. *Indicates that the monitored burrow entrance is likely to be inactive. Pas_1 was monitored on two separate occasions.

**Table:**

Burrow	Species	Dates monitored	#camera‐trap days	#porcupine records	#indiv in burrow	#species of other animals	Burrow type
Pas_1a	*Hystrix brachyura*	Dec 2019–Jan 2020	30	38	4 (2,1,1)	1	Rockface
Pas_1b	*Hystrix brachyura*	Jan 2021	27	10	3 (2,0,1)	0	Rockface
Pas_2	*Hystrix brachyura*	Feb–May 2020	72	124	4 (2,1,1)	4	Soil
Cad_1*	*Hystrix brachyura*	March–June 2020	64	1	1 (1,0,0)	4	Soil
Ek_1	*Hystrix brachyura*	March–April 2020	23	7	3 (2,0,1)	2	Soil
Day_1	*Hystrix brachyura*	Sept–Oct 2019	33	9	3 (2,0,1)	5	Soil
Day_3*	*Hystrix brachyura*	Nov 2019	11	0	0	3	Soil
Guh_1	*Atherus macrourus*	Jan 2019–March 2020	216	224	4 (2,1,1)	12	Rockface

## Appendix 2

Porcupine burrows in the Belum–Temenggor Forest Complex, Peninsular Malaysia: (Left) Soil burrow of the Malayan porcupine with fruit of *Callerya atropurpurea* (Fabaceae) left outside; (Right) Rockface burrow of the brush‐tailed porcupine.

## Appendix 3

Details of burrows used for measurements of seedling density and diversity.

**Table:**

Burrow code	Area searched on burrow (m)	Number of seedlings on burrow	Number of seedling species on burrow	Area searched away from burrow (m)	Number of seedlings away	Number of seedling species away from burrow
Day_1	9 × 1	48	18	5 × 2	14	6
Day_2	5 × 2	51	26	5 × 2	18	20
Day_3	10.4 × 1	36	22	10.4 × 1	29	20
Pla_1	4.5 × 1	11	11	10 × 1	16	11
Cad_1	6 × 1	24	15	8 × 1	32	16
Hal_1	4.4 × 2	26	19	10 × 1	32	20

## Appendix 4

Visits by animals to porcupine burrows, other than the resident porcupine. Number of burrows visited and number of records are listed as *Atherurus macrourus* /*Hystrix brachyura*. Animals are grouped as species that (1) enter and/or exit the burrow, (2) investigate the entrance, (3) appear to feed on insects outside the entrance.

**Table:**

Animal species	Common name or description	#burrows	#records
**(1) Enter/exit burrow**			
NA	Unidentified bats	1/1	> 8/> 13
*Maxomys* or *Rattus*	Unidentified medium‐sized rat	1/2	34/44
*Leopoldalmys*	Unidentified large rat/long tail	2/2	6/13
*Rhizomys pruinosus*	Indo‐Malayan bamboo rat	1/0	2/0
*Hystrix brachyura*	Malayan porcupine	1/0	1/0
Unidentified	Skink	1/0	1/0
*Tupaia glis*	Tree shrew	1/0	42/0
*Varanus* sp.	Monitor lizard	1/0	2/0
*Manis javanica*	Pangolin	0/1	0/2
*Urva urva*	Crab‐eating mongoose	0/1	0/1
**(2) Investigates entrance**			
*Canis familiaris*	Domestic dog	1/0	1/0
*Prionailurus bengalensis*	Leopard cat	1/0	1/0
*Urva urva*	Crab‐eating mongoose	0/1	0/1
*Callosciurus* sp.	Squirrel	0/1	0/1
*Muntiacus muntjak*	Barking deer	0/1	0/3
*Macaca leonina*	Pig‐tailed macaque	0/2	0/5
*Catopuma temminckii*	Asian golden cat	0/2	0/2
*Tragulus kanchil*	Mousedeer	0/1	0/1
**(3) Feeds insects**			
*Chalcophaps indica*	Emerald dove	1/0	3/0
Pittadae	Pitta	1/0	1/0
*Argusianus argus*	Great argus	0/2	0/3
Unidentified	Passerine	0/1	0/1
*Gallus gallus*	Junglefowl	0/1	0/2

## Appendix 5

List of plant species consumed by porcupines in the Belum–Temenggor forest complex identified through Local Ecological Knowledge. Shown are the fruit traits and handling behaviour.

**Table:**

Family	Species name	Fruit Type	Fruit width (mm)	Seed width (mm)	Fruit piercing	Seed hardness	Seed handling
Achariaceae	*Hydnocarpus sp 1*	Indehiscent fleshy berry	59.7	28.2	Difficult	Medium hard	Hoarded
Anacardiaceae	*Bouea oppositifolia*	Indehiscent fleshy berry	22.8	11.9	Easy	Medium hard	Dropped
Anacardiaceae	*Dracontomelon dao*	Indehiscent fleshy drupe	28.4	16.2	Moderate	Hard	Hoarded
Anacardiaceae	*Spondias cf malayana*	Indehiscent fleshy drupe	28.4	24.7	Easy	Hard	Hoarded
Annonaceae	*Alphonsea cf cylindrica*	Indehiscent fleshy berry	37.8	11.9	Moderate	Medium hard	Hoarded
Annonaceae	*Artabotrys sp*	Indehiscent fleshy drupe	20.8	11.9	Easy	Hard	Predated
Annonaceae	*Cyathocalyx sumatranus*	Indehiscent fleshy berry	55.7	19.2	Difficult	Hard	Hoarded
Annonaceae	*Meiogyne cf kanthanensis*	Indehiscent fleshy berry	18.1	7.4	Moderate	Medium hard	Predated
Annonaceae	*Monocarpia maingayi*	Indehiscent fleshy berry	63.2	19.8	Difficult	Medium hard	Predated
Annonaceae	*Monoon cf lateriflorum*	Indehiscent fleshy‐dry drupe	10.6	5.8	Easy	Medium hard	Predated
Annonaceae	*Platymitra macrocarpa*	Indehiscent fleshy berry	41.1	15.8	Moderate	Medium hard	Hoarded
Annonaceae	*Stelechocarpus cauliflorus*	Indehiscent fleshy berry	31.3	10.4	Moderate	Medium hard	Predated
Annonaceae	*Uvaria grandiflora*	Indehiscent fleshy berry	12.5	8.1	Easy	Medium hard	Predated
Apocynaceae	*Willughbeia cf flavescens*	Indehiscent fleshy berry	82.7	19.9	Difficult	Medium hard	Hoarded
Arecaceae	*Eugeissona tristis*	Indehiscent fleshy drupe	42.1	36.9	Difficult	Hard	Hoarded
Burseraceae	*Canarium cf asperum*	Indehiscent fleshy drupe	13.6	7.7	Easy	Hard	Predated
Burseraceae	*Santiria cf laevigata*	Indehiscent fleshy drupe	14.4	8.6	Easy	Soft	Predated
Calophyllaceae	*Calophyllum macrocarpum*	Indehiscent fleshy drupe	55.0	31.5	Moderate	Hard	Predated
Calophyllaceae	*Mesua ferrea*	Dehiscent dry capsule	29.1	19.4	Difficult	Hard	Predated
Celastraceae	*Salacia sp*	Indehiscent fleshy berry	32.4	15.3	Difficult	Medium	Predated
Centroplacaceae	*Bhesa cf robusta*	Indehiscent fleshy drupe	5.8	5.0	Easy	Hard	Predated
Chrysobalanaceae	*Atuna excelsa*	Indehiscent dry nut	41.5	25.7	Difficult	Medium	Predated
Chrysobalanaceae	*Parinari rigida*	Indehiscent fleshy drupe	42.5	46.9	Easy	Hard	Predated
Clusiaceae	*Garcinia cf cantleyana*	Indehiscent fleshy berry	58.5	27.5	Moderate	Hard	Predated
Clusiaceae	*Garcinia cf forbesii*	Indehiscent fleshy berry	51.0	8.2	Moderate	Soft medium	Predated
Clusiaceae	*Garcinia cf xanthochymus*	Indehiscent fleshy berry	65.5	25.7	Easy	Medium hard	Predated
Clusiaceae	*Garcinia parvifolia*	Indehiscent fleshy berry	17.1	5.4	Easy	Soft medium	Predated
Connaraceae	*Connarus cf paniculatus*	Dehiscent fleshy follicle	25.5	10.5	Moderate	Medium	Predated
Cornaceae	*Alangium cf ebenaceum*	Indehiscent fleshy drupe	18.6	13.9	Easy	Medium hard	Hoarded
Ebenaceae	*Diospyros confertiflora*	Indehiscent fleshy drupe	10.8	7.4	Easy	Medium	Predated
Euphorbiaceae	*Balakata cf baccata*	Indehiscent fleshy drupe	14.1	4.5	Easy	Medium hard	Predated
Euphorbiaceae	*Elateriospermum tapos*	Dehiscent dry capsule	40.7	19.1	Difficult	Hard	Hoarded
Euphorbiaceae	*Paracroton pendulus*	Dehiscent fleshy capsule	32.0	11.5	Easy	Medium	Predated
Fabaceae	*Adinobotrys atropurpureus*	Dehiscent dry pod	56.2	41.7	Difficult	Medium hard	Hoarded
Fabaceae	*Intsia palembanica*	Dehiscent dry pod	80.2	24.9	Moderate	Medium	Hoarded
Fabaceae	*Saraca cf indica*	Dehiscent dry pod	70.1	26.8	Moderate	Medium	Hoarded
Fabaceae	*Sindora cf coriacea*	Dehiscent fleshy follicle	41.8	15.5	Moderate	Medium	Predated
Fagaceae	*Castanopsis corallocarpus*	Indehiscent dry nut	33.5	23.6	Difficult	Hard	Predated
Fagaceae	*Lithocarpus elegans*	Indehiscent dry nut	18.3	11.1	Difficult	Hard	Predated
Fagaceae	*Lithocarpus leptogyne*	Indehiscent dry nut	18.2	10.6	Difficult	Hard	Predated
Fagaceae	*Lithocarpus macphailii*	Indehiscent dry nut	19.0	13.0	Difficult	Hard	Predated
Fagaceae	*Lithocarpus rassa*	Indehiscent dry nut	18.3	11.1	Difficult	Hard	Predated
Fagaceae	*Lithocarpus suffruticosus*	Indehiscent dry nut	16.8	9.7	Difficult	Hard	Predated
Fagaceae	*Lithocarpus sundaicus*	Indehiscent dry nut	17.4	11.9	Difficult	Hard	Predated
Fagaceae	*Quercus oidocarpa*	Indehiscent dry nut	20.2	13.5	Difficult	Hard	Predated
Gnetaceae	*Gnetum cf cuspidatum*	Indehiscent fleshy drupe	14.0	8.7	Moderate	Hard	Predated
Gnetaceae	*Gnetum cf latifolium*	Indehiscent fleshy drupe	13.7	10.5	Moderate	Hard	Predated
Gnetaceae	*Gnetum cf microcarpum*	Indehiscent fleshy drupe	9.6	8.5	Moderate	Hard	Predated
Irvingiaceae	*Irvingia malayana*	Indehiscent fleshy drupe	40.2	28.2	Moderate	Hard	Hoarded
Lauraceae	*Cryptocarya cf densiflora*	Indehiscent fleshy drupe	19.2	11.7	Easy	Medium	Predated
Magnoliaceae	*Magnolia champaca*	Dehiscent dry follicle	16.5	12.4	Moderate	Hard	Predated
Malvaceae	*Heritiera javanica*	Indehiscent dry samara	13.6	10.0	Moderate	Soft	Predated
Malvaceae	*Sterculia monosperma var. monosperma*	Dehiscent dry follicle	34.2	12.8	Difficult	Soft medium	Hoarded
Meliaceae	*Aglaia cf elaeagnoidea*	Indehiscent fleshy drupe	10.7	8.1	Easy	Medium hard	Predated
Meliaceae	*Aglaia cf leucophylla*	Indehiscent fleshy drupe	14.4	10.8	Easy	Medium	NA
Meliaceae	*Chisocheton cf macrophyllus*	Indehiscent fleshy berry	65.2	24.9	Moderate	Medium hard	Hoarded
Meliaceae	*Sandoricum cf beccarianum*	Indehiscent fleshy berry	41.0	10.8	Moderate	Medium hard	Hoarded
Moraceae	*Artocarpus elasticus*	Indehiscent fleshy multiple	96.5	8.8	Difficult	Medium	Predated
Moraceae	*Artocarpus lanceifolius*	Indehiscent fleshy multiple	115.7	10.6	Difficult	Medium	Predated
Moraceae	*Artocarpus rigidus*	Indehiscent fleshy multiple	94.4	9.9	Difficult	Medium	Predated
Moraceae	*Ficus cf acamptophylla*	Indehiscent fleshy syconium	7.9	0.8	Easy	Medium	Defecated
Moraceae	*Ficus delosyce*	Indehiscent fleshy syconium	8.8	0.7	Easy	Medium	Defecated
Moraceae	*Ficus scaberrima*	Indehiscent fleshy syconium	24.3	1.1	Moderate	Medium hard	Defecated
Moraceae	*Ficus subgelderi*	Indehiscent fleshy syconium	12.0	0.8	Easy	Medium	Defecated
Moraceae	*Ficus sundaica*	Indehiscent fleshy syconium	11.4	0.8	Easy	Medium	Defecated
Myristicaceae	*Myristica cf elliptica*	Dehiscent fleshy follicle	22.5	16.9	Difficult	Hard	Predated
Myristicaceae	*Myristica cinnamomea*	Dehiscent fleshy follicle	41.1	21.9	Difficult	Hard	Predated
Myrtaceae	*Syzygium cf duthieanum*	Indehiscent fleshy drupe	10.5	5.8	Easy	Medium hard	Predated
Myrtaceae	*Syzygium muelleri*	Indehiscent fleshy drupe	10.8	8.2	Easy	Hard	Predated
Olacaceae	*Scorodocarpus Borneensis*	Indehiscent fleshy drupe	39.2	38.0	Difficult	Hard	Hoarded
Rhamnaceae	*Ziziphus cf oenopolia*	Indehiscent fleshy drupe	9.3	6.9	Easy	Hard	Predated
Rubiaceae	*Catunaregam cf oocarpa*	Indehiscent fleshy pepo	58.6	12.1	Moderate	Soft	Hoarded
Rubiaceae	*unid_Chenok*	Indehiscent fleshy drupe	20.4	13.2	Easy	Hard	Hoarded
Rutaceae	*Citrus hystrix*	Indehiscent fleshy hesperidium	89.7	8.0	Difficult	Medium	Dropped
Sapindaceae	*Pometia pinnata*	Indehiscent fleshy drupe	15.5	8.8	Moderate	Medium	Predated
Sapindaceae	*Xerospermum noronhianum*	Indehiscent fleshy drupe	17.3	12.2	Moderate	Medium hard	Predated
Styracaceae	*Styrax benzoin*	Indehiscent fleshy drupe	24.8	14.2	Moderate	Hard	Predated
Unidentified	*unid_Cenos*	Indehiscent fleshy drupe	8.6	7.0	Easy	Medium	Predated
Unidentified	*unid_Jerantok*	Indehiscent fleshy berry	32.0	13.0	Moderate	Medium	Predated
Unidentified	*unid_Tahbit*	Indehiscent fleshy drupe	10.4	8.1	Easy	Medium hard	Hoarded

## Appendix 6

Camera‐trap results at a tree of *Callerya atropurpurea* (Fabaceae). Camera was set out for 16 days (1 September 2019 to 17 September 2019). Rats, muntjac and a civet passed by without interacting with the fruits and are not noted here.

**Table:**

Species	Date	Time	Visit length (min)	#Indiv.	Observations
*Hystrix brachyura*	1 Sept	21:55	1	1	Removes 1 fruit
*Hystrix brachyura*	1 Sept	22:10	7	2+	Removes > 1 fruit
*Hystrix brachyura*	3 Sept	02:14	11	2+	Removes ~9 fruits
*Hystrix brachyura*	3 Sept	22:56	6	2	Removes 2 fruits
*Hystrix brachyura*	5 Sept	01:00	1	2	Removes 1 fruit
*Hystrix brachyura*	5 Sept	01:50	1	1	Removes 1 fruit
*Hystrix brachyura*	7 Sept	00:02	15	2	Removes 2 fruit
*Hystrix brachyura*	7 Sept	00:49	1	2	Removes 1 fruit
*Hystrix brachyura*	7 Sept	23:19	2	2	Removes 3 fruits
*Hystrix brachyura*	8 Sept	21:58	91	2	Removes 8 fruits
*Hystrix brachyura*	9 Sept	22:23	1	1	No interaction
*Hystrix brachyura*	10 Sept	03:56	1	1	Removes 1 fruit
*Hystrix brachyura*	11 Sept	02:26	3	2	Removes 4 fruits
*Hystrix brachyura*	12 Sept	23:01	18	1	Removes 2 fruits
*Hystrix brachyura*	15 Sept	04:03	39	2	Removes 4 fruits; search time is long as fruiting stops.
*Elephas maximus*	17 Sept	00:13	1	1	Cannot see what the animal is doing
